# P02.08. Patient reported outcomes from complementary, alternative, and integrative medicine (PROCAIM): a feasibility practice network study

**DOI:** 10.1186/1472-6882-12-S1-P64

**Published:** 2012-06-12

**Authors:** D Ackerman, L Shinto, M Hardy, A Heyman, L Nguyen, A Senders, D Abrams, A Ali, B Enebo, P Homel, R Horowitz, M Reardon, C Torkelson

**Affiliations:** 1 Oregon College of Oriental Medicine, Portland, USA; 2 Oregon Health & Science University, Portland, USA; 3 University of California, Los Angeles, Los Angeles, USA; 4 University of Michigan, Ann Arbor, USA; 5 National College of Natural Medicine, Portland, USA; 6 University of California, San Francisco, Osher Center, San Francisco, USA; 7 Yale University, Integrative Medicine Center at Griffin Hosptial, Derby, USA; 8 University of Colorado School of Medicine, Denver, USA; 9 Albert Einstein College of Medicine, Bronx, USA; 10 University of Arizona, Center for Integrative Medicine, Tucson, USA; 11 University of Vermont College of Medicine, Burlington, USA; 12 University of Minnesota, Minneapolis, USA

## Purpose

To assess the feasibility of utilizing a web-based data collection system as the research infrastructure for conducting multi-site outcomes research to evaluate complementary, alternative, and integrative medicine.

## Methods

The study was a longitudinal observational, cohort study with data collected at baseline and three months. Nine integrative medicine clinics housed at academic health centers that were members of the Consortium for Academic Health Centers for Integrative Medicine (CAHCIM) were invited to participate. Each academic health center was asked to obtain IRB approval for the study. Clinic patients enrolled in the study were given instructions for logging on to the project website to complete the following questionnaires: demographic, quality of life (SF-12), brief pain inventory, and an evaluation of the web-based system. Three months after completing baseline questionnaires patients were notified by email with instructions to complete final quality of life and pain questionnaires. Change in quality of life and pain were analyzed by paired t-test.

## Results

All nine centers obtained IRB approval and subjects gave informed consent. Eighty patients from eight centers were enrolled and 48% (38/80) completed the baseline questionnaires. Of those that completed baseline questionnaires, 58% (22/38) completed the 3-month questionnaires. Eighty-four percent (32/38) at baseline completed the evaluation rating their experience with the system and 74% (28/38) responded that the web-based system was easy to navigate. Only 16 participants completed both baseline and 3-month quality of life and pain questionnaires, limiting interpretation of this data. Significant improvements in the mental health component of SF-12 (MCS, p=0.034), pain severity (p=0.02) and pain interference (p=0.01) were observed.

## Conclusion

The study shows the feasibility of web-based data collection from multiple sites. Limited study resources likely contributed to a significant number of enrolled participants not completing surveys. Although limited, the data showing improvements in MCS and pain warrant further investigation.

